# Maternal Obesity Is Associated with Alterations in the Gut Microbiome in Toddlers

**DOI:** 10.1371/journal.pone.0113026

**Published:** 2014-11-19

**Authors:** Jeffrey D. Galley, Michael Bailey, Claire Kamp Dush, Sarah Schoppe-Sullivan, Lisa M. Christian

**Affiliations:** 1 Division of Biosciences, College of Dentistry, Ohio State University, Columbus, Ohio, United States of America; 2 The Institute for Behavioral Medicine Research, The Ohio State University Wexner Medical Center, Columbus, Ohio, United States of America; 3 Department of Human Science, The Ohio State University, Columbus, Ohio, United States of America; 4 Department of Psychiatry, The Ohio State University Wexner Medical Center, Columbus, Ohio, United States of America; 5 Department of Obstetrics and Gynecology, The Ohio State University Wexner Medical Center, Columbus, Ohio, United States of America; 6 Psychology, The Ohio State University, Columbus, Ohio, United States of America; University of Arkansas for Medical Sciences, United States of America

## Abstract

Children born to obese mothers are at increased risk for obesity, but the mechanisms behind this association are not fully delineated. A novel possible pathway linking maternal and child weight is the transmission of obesogenic microbes from mother to child. The current study examined whether maternal obesity was associated with differences in the composition of the gut microbiome in children in early life. Fecal samples from children 18–27 months of age (n = 77) were analyzed by pyro-tag 16S sequencing. Significant effects of maternal obesity on the composition of the gut microbiome of offspring were observed among dyads of higher socioeconomic status (SES). In the higher SES group (n = 47), children of obese (BMI≥30) versus non-obese mothers clustered on a principle coordinate analysis (PCoA) and exhibited greater homogeneity in the composition of their gut microbiomes as well as greater alpha diversity as indicated by the Shannon Diversity Index, and measures of richness and evenness. Also in the higher SES group, children born to obese versus non-obese mothers had differences in abundances of *Faecalibacterium* spp., *Eubacterium* spp., *Oscillibacter* spp., and *Blautia* spp. Prior studies have linked some of these bacterial groups to differences in weight and diet. This study provides novel evidence that maternal obesity is associated with differences in the gut microbiome in children in early life, particularly among those of higher SES. Among obese adults, the relative contribution of genetic versus behavioral factors may differ based on SES. Consequently, the extent to which maternal obesity confers measureable changes to the gut microbiome of offspring may differ based on the etiology of maternal obesity. Continued research is needed to examine this question as well as the relevance of the observed differences in gut microbiome composition for weight trajectory over the life course.

## Introduction

Obesity is a substantial public health problem globally. In the US, it is estimated that 16.9% of children ages 2–19 years and 33.8% of adults ≥20 years are obese [1], [2]. However, early life antecedents of obesity are not well delineated. In children under 3 years of age, the strongest predictor of obesity in adolescence and adulthood is parental obesity [3]. Compared to paternal obesity, maternal obesity has greater predictive value for body mass index (BMI) of offspring through adolescence [4], [5]. However, the relative influence of genetics versus environmental pathways in the transgenerational transmission of obesity from parent to child is unknown.

A novel possible mechanistic pathway linking parental and child weight is the transmission of commensal microbiota via parental exposures, particularly maternal. The microbiota are a consortium of trillions of bacteria that are resident to a variety of human body niches [6]. The vast majority of these microbes reside within the gastrointestinal (GI) tract where they form microbial communities whose structures are stable during periods of homeostasis and heavily involved in host metabolic and nutritional functions, including food digestion and vitamin synthesis [7], [8].

Disruptions in the relative abundances of microbes that comprise these communities have been associated with obesity and high-fat diets [9]–[14]. For example, obese mice have abnormal levels of GI *Firmicutes* and *Bacteroidetes*, two primary phyla of the GI tract microbiota [12]. Such skewed bacterial abundances may lead to alterations in energy procurement from food and related propensity toward obesity. When microbiota from obese mice are transferred into germ-free mice, recipient mice have increased body fat, providing strong evidence of a causal link between the microbiota and obesity [14].

Factors affecting the establishment of bacterial abundances in early life are not well understood. During birth, the neonate is rapidly colonized by maternal bacteria via vertical transmission from the gastrointestinal and reproductive tracts as well as environmental microbes [15]–[17]. In very early life, mothers are likely to be primary donors of bacteria through physical contact and breast milk. Demonstrating such maternal influence, at one and six months of age, infants of obese mothers have significantly different bacterial population abundances compared to infants of non-obese mothers [18]. Importantly, during the first year of life, the microbiota show great transience and volatility [19]. As solid foods are introduced to the diet, the structure of the microbiota stabilizes and begins to reflect the adult profile [20]. Thus, it is important to determine if maternal influences on gut microbial groups persist in children past early infancy despite competing factors.

In addition, the recent advent of next generation pyrosequencing allows for wider study of microbial communities than permitted by earlier methods, including denaturing gradient gel electrophoresis (DGGE) and polymerase chain reaction (PCR). Utilization of this technology permits the analyses of entire bacterial communities rather than examination of smaller classification subsets selected by *a priori* hypotheses. To our knowledge, pyrosequencing has not been used in studies associating parental obesity to child microbiota communities.

Addressing these gaps in the literature, the current study examined the association between maternal obesity and the gut microbiota profiles of toddlers at approximately two years of age using pyrosequencing technology. We hypothesized that the microbiota of children born to obese mothers would have a significantly different gastrointestinal microbiota, as assessed using alpha and beta diversity measurements, when compared to children born to normal weight mothers. We also hypothesized that differences in abundances of bacterial populations previously associated with obesity would be observed in children of obese versus non-obese mothers.

## Methods

### Study Design

We recruited 79 women with children approximately two years of age from the general community of Columbus, Ohio. Children were excluded if their mother reported the child had a major health condition or developmental delay. Children were also excluded if they were already toilet trained. Each woman completed an online questionnaire which included assessment of her health behaviors and exposures (e.g., medications) during pregnancy as well as health and feeding behaviors in her child.

Within 7 days of completing the online questionnaire, each woman collected a stool sample from her child per the protocol detailed below. Two samples were removed from statistical analyses due to low sequence count (<5108), resulting in final sample of 77 mother-child pairs. This study was approved by the Ohio State University Biomedical Institutional Review Board. All women completed written informed consent for themselves and provided written consent on behalf of their children. Women received modest compensation for their participation. Data collection occurred from May 2011 to December 2012.

### Parental Characteristics

Women reported information about their age, race (self and child’s father), marital status, education level (self and child’s father), and total family income per year. Body mass index (BMI; kg/m^2^) was calculated based on the provided maternal and paternal heights and weights. BMI values ≥30 were classified as obese.

### Perinatal Health Information

Self-report data was collected regarding exposure to antibiotics during pregnancy and while breastfeeding (if applicable). With regard to birth outcomes, women reported the route of delivery (vaginal versus C-section), gestational age at the time of delivery and the child’s sex.

### Child Diet and Growth

Women reported the occurrence and duration of breastfeeding and the age at which formula (if applicable), cereals/grains, fruits/vegetables, and meats were introduced as part of the child’s diet. The current frequency of each food type was also reported, from less than once per month to two or more times per day. Women reported the number of times their child had been exposed to antibiotic medications, with completion of a full prescription course (e.g., 10 days) considered as one exposure. Women also reported child exposure to probiotics in capsule/supplement form or in formula or food which specified it contained probiotics.

Finally, to determine the child’s growth trajectory, women reported their child’s height and weight percentile at the most recent well-visit to the pediatrician. A weight/height ratio was calculated and children were categorized into three groups: those whose weight percentile was greater than their height percentile (n = 11), those in the same percentile bracket (n = 31), and those whose weight percentile was lower than their height percentile (n = 33).

### Stool Sample Collection and Storage

Women were provided with sterile wooden applicators and sterile 50 ml plastic conical collection tubes for collection. They were instructed to sterilely collect the stool sample from child’s soiled diaper with the wooden applicator and place in the collection tube. Samples were then stored at 4°C (i.e., refrigerated) for up to 24 hours until collection by study personnel from the participant’s home or delivery by the participant to OSUWMC. In the latter case, women were instructed to transport samples in a cooler with ice. Upon arrival at the Wexner Medical Center, samples were placed in long-term storage at −80°C until pyrosequencing was conducted.

### bTEFAP

Bacterial tag-encoded FLX-Amplicon Pyrosequencing (bTEFAP) was performed as previously described [21], [22]. The 16 s rrn universal primers 27f (AGA GTT TGA TCM TGG CTC AG) and 519r (GWATTACCGCGGCKGCTG) were used for specific 16S rrn targeting.

These primers were used for single-step 30 cycle PCR. The following thermoprofile was used: a single cycle of 94°C for 3 minutes, then 28 cycles of: 30 seconds at 94°C; 40 seconds at 53°C, 1 minute at 72°C, with a single 5 minute cycle at 72°C for 5 minutes for elongation. Amplicons were pooled at equivalent concentrations and purified (Agencourt Bioscience Corporation, MA, USA). Sequencing was performed with the Roche 454 FLX Titanium system using manufacturer’s guidelines.

### Sequencing Analysis

Analysis was performed using the open-source software package, Quantitative Insights Into Microbial Ecology (QIIME), v.1.7.0. [23]. Sequences were provided via.fasta file and sequence quality was denoted with a.qual file. Barcodes were trimmed and low-quality reads were removed. An average quality score of 25 was used. Minimum sequence length of 200 and maximum length of 1000 were used. No mismatches were allowed in the primer sequence. An average of 14862 sequences were attained per sample, and a total of 77.06% of sequences passed quality filtering.

Sequences were clustered based upon 0.97 similarity using UClust into operational taxonomic units (OTUs) [24]. A representative sequence was selected from each OTU and the RDP classifier was used to assign taxonomy to the representative sequence [25]. Sequences were aligned using PyNAST [26] against a Greengenes core reference alignment database [27] and an OTU phylogenetic tree was assembled based upon this alignment [28].

Phylogenetic Investigation of Communities by Reconstruction of Unobserved States, or PiCRUST, was used to identify differences in predictive metagenome function [29]. In summary, OTUs were picked from a demultiplexed fasta file containing the sequences for all 77 subjects using the closed-reference procedure, against the GreenGenes 13_5 reference database [30]. These OTUs were normalized by the predicted 16 s copy number, and functions were predicted from these normalized OTUs with the use of GreenGenes 13_5 database for KEGG Orthologs. From this, a BIOM table containing the predicted metagenome for each sample was attained. Each sample was rarefied at 2,000,000 before further analysis. Downstream statistical analysis was performed using STAMP [31].

### Statistical Analysis

The Shannon Diversity Index (SDI), a measurement of within-sample (alpha-diversity) community diversity, as well as Chao1 (estimates richness), equitability (measures evenness), and observed_species (calculates unique OTUs) were used to ascertain differences in alpha diversity based on maternal obesity status [32]. All alpha-diversity measurements were calculated with QIIME and significance was measured using a parametric t-test at a depth of 5930 sequences for comparison of all obese vs non-obese groups. Depths of 4534 sequences for comparison of maternal obesity among the high income group alone, and 5126 sequences for comparison among the low income group alone were also used. UniFrac unweighted distance matrices were calculated from the OTU phylogenetic tree for beta diversity analyses [33]. A sampling depth of 5108 sequences/sample was used for beta diversity for all groups.

The adonis statistic, available through the vegan package on the open-source statistical program R, and further employed in QIIME, was used to measure differences in variance between two groups based upon their microbiota UniFrac distance matrices [34], [35]. Groups were split based upon maternal and paternal BMI, as well as by income level and differences in community structure were determined using adonis. The permdisp statistic, also available through vegan, was then performed to verify equal variances between groups dichotomized by obesity.

Chi-square analyses and two-sample t-tests were used to determine the demographic and behavioral similarity between the maternal obesity groups to identify possible confounding factors. Additionally, Pearson’s correlations, univariate analysis of variance (ANOVA) and regression analyses were used to examine associations between variables including maternal BMI, child’s weight/height ratio and the SDI. The relative abundance of bacterial groups in samples from children of obese and non-obese mothers were compared using Mann-Whitney U-tests. All analyses were performed using SPSS v.21 (IBM, Chicago, IL). For predictive functional group analysis in STAMP, Welch’s t-tests were used for two group comparisons, while Kruskal-Wallis H-tests were used for multiple group comparisons. P-values were corrected for multiple-tests using the Benjamini-Hochberg method [36], with a q-value of 0.10.

## Results

### Participant Characteristics

This study included 77 mother-child pairs. Children were 18–27 months at the time of assessment (Mean = 23.14, SD = 2.00), with 91% falling between 21–26 months. In this sample, 87.0% (n = 67) of mothers were White, 9.1% (n = 7) were Black and 3.9% (n = 3) were Asian. The mean maternal age at the time of delivery was 31.10±5.43 and 87.0% of women (n = 67) were married. In this sample, 66.2% of mothers (n = 51) were non-obese (BMI <30) and 33.8% (n = 26) were obese (BMI ≥30) based self-reported height and weight prior to pregnancy. The mean BMI among the obese women was 35.13±4.48 compared to 22.65±2.85 among the non-obese (t(75) = 15.3, *p*<0.001).

To identify potential factors which may confound the relationship between maternal obesity and the composition of the child microbiome, we examined the demographic and behavioral similarity between obese and non-obese women (Tables 1 & 2). Obese and non-obese women did not differ significantly in race, marital status, maternal age at the time of delivery, antibiotic exposure during pregnancy or breastfeeding, or delivery route (vaginal versus C-section). Obese women had heavier male partners than did non-obese women, with BMIs of 31.20±5.98 vs. 26.91±4.60, respectively (t(75) = 3.49, *p* = 0.001). Obese women and their partners had completed less education than non-obese women and their partners (*ps* ≤0.014). However, women did not differ in annual household income based on obesity status (X^2^(3) = 1.92, *p* = .59), although household income was significantly correlated with both maternal (r = .65, *p*<0.001) and paternal education (r = .52, *p*<0.001).

**Table 1:** Demographic Characteristics.

	Total(n = 77)	Obese(n = 26)	Non-Obese(n = 51)	Obese vs.Non-Obese
**Maternal BMI** [Mean (SD)]	26.86 (6.83)	35.13 (4.48)	22.65 (2.85)	t(75) = 15.3, *p* = .000*
**Paternal BMI** [Mean (SD)]	28.34(5.47)	31.2 (5.98)	26.89 (4.92)	t(75) = 3.49, *p* = .001*
**Maternal Age** [Mean (SD)]	31.10 (5.43)	31.96 (6.02)	30.67 (5.11)	t(75) = 0.99, *p = *.33
**Child Sex** [n (%)]				X^2^(1) = 0.79, *p* = .37
Male	41 (53.2%)	12 (46.2%)	29 (56.9%)	
Female	36 (46.8%)	14 (53.8%)	22 (43.1%)	
**Maternal Race**				X^2^(1) = 0.47, p = .47@
White	67 (87.0%)	21 (80.8%)	46 (90.2%)	
Black/African-American	7 (9.1%)	5 (19.2%)	2 (3.9%)	
Asian	3 (3.9%)	0 (0%)	3 (5.9%)	
**Marital Status** [n (%)]				X^2^(1) = 3.54, *p* = .06
Married	67 (87.0%)	20 (76.9%)	47 (92.2%)	
Unmarried	10 (13%)	6 (23.1%)	4 (7.8%)	
**Maternal Education** [n (%)]				X^2^(2) = 10.67^‡^, *p* = .005*
High school graduate or less	19 (24.7%)	12 (46.2%)	7 (13.7%)	
College graduate (2 or 4 yr)	26 (33.8%)	8 (30.8%)	18 (35.3%)	
Some graduate school or higher	32 (41.6%)	6 (23.1%)	26 (51.0%)	
**Paternal Education** [n (%)]				X^2^(2) = 8.51, *p* = .014*
High school graduate or less	29 (37.6%)	12 (46.2%)	17 (33.3%)	
College graduate (2 or 4 yr)	30 (39.0%)	12 (46.2%)	18 (35.3%)	
Some graduate school or higher	18 (23.3%)	2 (7.7%)	16 (31.4%)	
**Income** [n (%)]				X^2^(3) = 1.92, *p* = .59
< $ 30,000	15 (19.5%)	7 (26.9%)	8 (15.7%)	
$30,000–49,999	15 (19.5%)	5 (19.2%)	10 (19.6%)	
$50,000–99,999	30 (39.0%)	10 (38.4%)	20 (39.2%)	
≥ $100,000	17 (22.0%)	4 (15.4%)	13 (25.5%)	

*p<.05.

@White versus non-white.

**Table 2:** Health/Behavioral Characteristics.

	Total(n = 77)	Obese(n = 26)	Non-Obese(n = 51)	Obese vs.Non-Obese
**Route of delivery** [n (%)]				X2(1) = 0.17, p = .68
C-Section	33 (42.9%)	12 (46.2%)	21 (41.2%)	
Vaginal	44 (57.1%)	14 (53.8%)	30 (58.8%)	
**Breastfeeding duration** [n (%)]				X^2^(2) = 3.84, *p* = .147^#^
Never	5 (6.5%)	4 (15.4%)	1 (2%)	
<3 months	7 (9.1%)	3 (11.5%)	4 (7.8%)	
3 to 11 months	38 (49.4%)	11 (42.3%)	27 (52.9%)	
≥12 months	27 (35.1%)	8 (30.8%)	19 (37.2%)	
**Grains/Cereals introduced** [n (%)]				X^2^(1) = 0.06, *p* = .812∧
≤4 months	30 (39.0%)	12 (46.2%)	18 (35.3%)	
5−6 months	41 (53.2%)	11 (42.3%)	30 (58.8%)	
≥7 months	6 (7.8%)	3 (11.5%)	3 (5.9%)	
**Vegetables, fruits, and/or meats introduced** [n (%)]				X^2^(2) = 0.17, *p* = .92
≤4 months	17 (22.1%)	6 (23.1%)	11 (21.6%)	
5−6 months	38 (49.4%)	12 (46.2%)	26 (51.0%)	
≥7 months	22 (28.6%)	8 (30.8%)	14 (27.5%)	
**Meat frequency** [n (%)]				X^2^(2) = 2.24, *p* = .33
Less than once per day	25 (32.5%)	6 (23.1%)	19 (37.3%)	
Once per day	27 (35.1%)	9 (34.6%)	18 (35.3%)	
More than once per day	25 (32.5%)	11 (42.3%)	32 (27.5%)	
**Vegetable frequency** [n (%)]				X^2^(2) = 0.30, *p* = .86
Less than once per day	17 (22.1%)	5 (19.2%)	12 (23.5%)	
Once per day	24 (31.2%)	9 (34.6%)	15 (29.4%)	
More than once per day	36 (46.8%)	12 (46.2%)	24 (47.1%)	
**Antibiotic use in pregnancy** [n (%)]				X2(1) = 1.07, *p* = .30
No	64 (83.1%)	20 (76.9%)	44 (86.3%)	
Yes	13 (16.9%)	6 (23.1%)	7 (13.7%)	
**Antibiotic use while breastfeeding** [n (%)]				X2(1) = .056, *p* = .81
No	69 (89.6%)	23 (88.5%)	46 (90.2%)	
Yes	8 (10.4%)	3 (11.5%)	5 (9.8%)	
**Antibiotic exposure in child** [n (%)]				X2(2) = 2.30, *p* = .317
None	23 (29.9%)	5 (19.2%)	18 (35.3%)	
One or two courses	29 (37.7%)	12 (46.2%)	17 (33.3%)	
More than two courses	25 (32.4%)	9 (34.6%)	16 (31.4%)	

#Never and <3 months combined in analyses due to low occurrence.

∧ 5–6 months and ≥7 months combined in analyses due to low occurrence.

### Maternal obesity and beta diversity in the child gut microbiome

**Figure 1:**
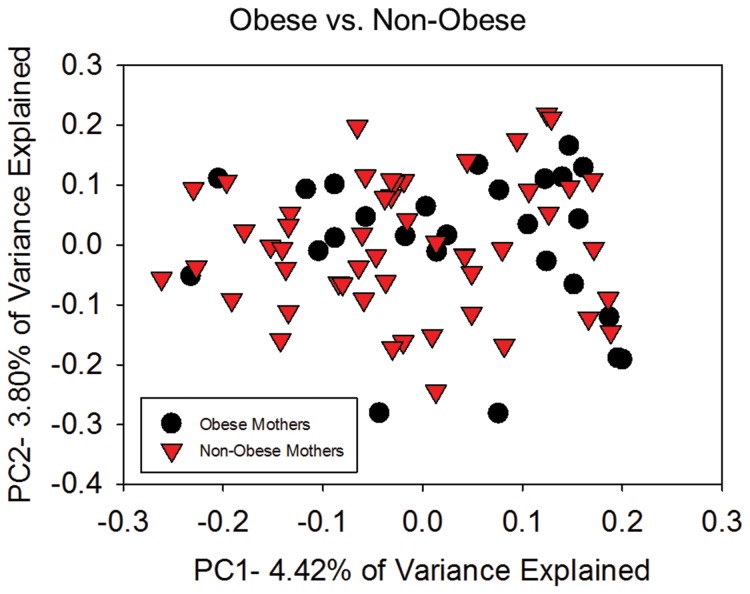
In the overall sample, datapoints did not cluster on a principle coordinate analysis (PCoA) scatter-plot as a function of maternal obesity. The beta-diversity non-parametric statistic adonis showed that children born to obese (n = 26) versus non-obese mothers (n = 51) had unique microbial profiles (*p* = 0.044). However, this was due to greater homogeneity among the obese group as measured with permdisp (*p* = 0.035).

Unweighted UniFrac distance matrices were used to assess differences between the microbial communities, known as beta diversity, in children of obese compared to non-obese mothers. Permutational multivariate ANOVA using adonis showed that children of obese versus non-obese mothers had a different microbiota community structure (*r* ^2^ = 0.01539, *p* = 0.044). However, this did not result in clustering of two distinct populations using a principle coordinate analysis (PCoA) (Fig. 1). To further explain the significant adonis statistic in the absence of obvious clustering, permdisp, a statistic that measures the extent to which variances in different populations are equivalent, was used to compare the two groups. Dispersion of the community structures of children born to obese versus non-obese mothers differed signficantly, with greater variance among children of non-obese mothers (*p* = 0.035, *F* = 4.843). In contrast, there was no difference in between-sample community structure as measured via adonis in children of obese versus non-obese fathers (*r* ^2^ = 0.01214, *p* = 0.801).

**Figure 2:**
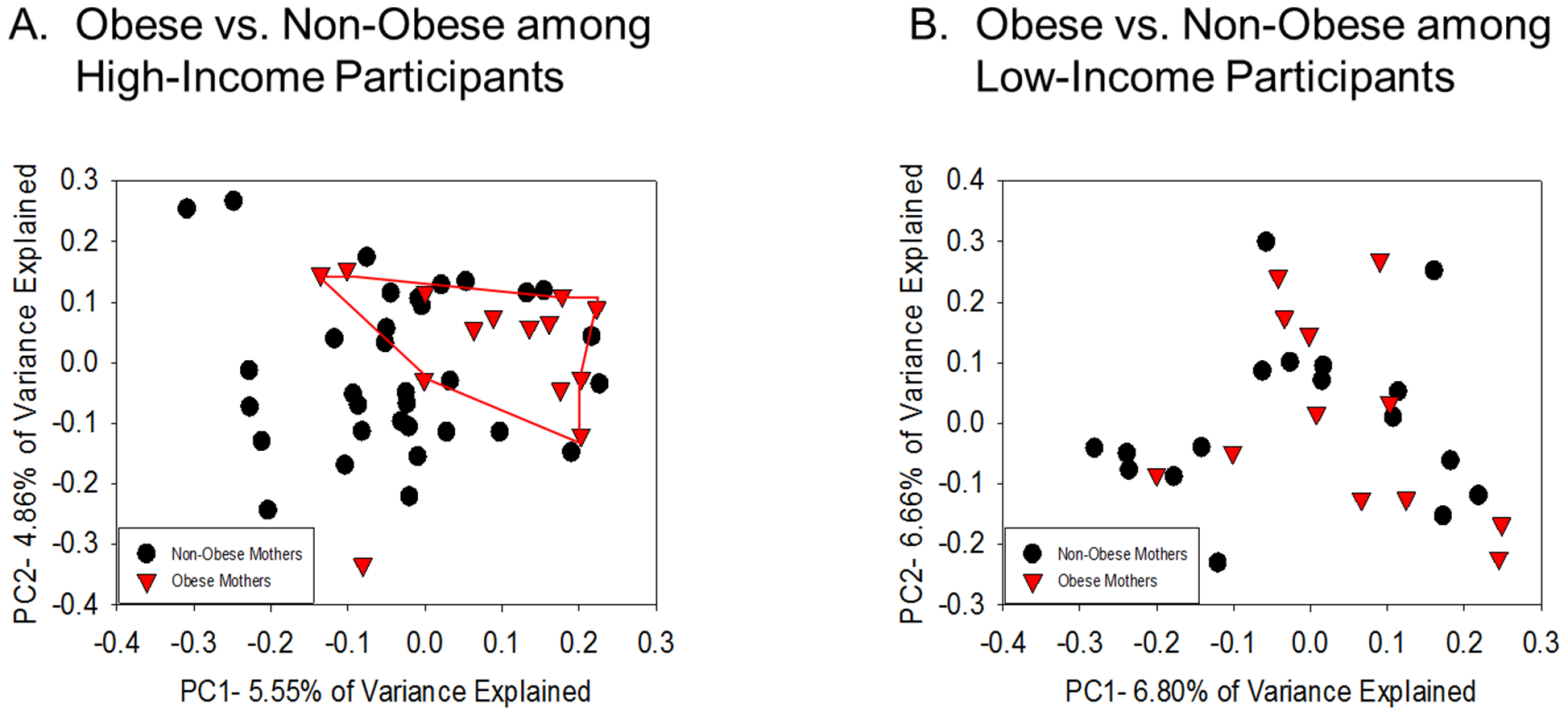
Interactive effects of maternal obesity and socioeconomic status were observed; effects of maternal obesity on the child microbiome were primarily seen among the higher SES group. A) In the higher income group, children born to obese versus non-obese mothers clustered (adonis, p = 0.041) and had higher homogeneity (permdisp, p = 0.003). B) These effects of maternal obesity were not seen in children in the lower income group.

Next, we examined whether the strength of the association between beta diversity and maternal obesity differed among children of mothers from higher versus lower socioeconomic backgrounds. Analyses showed no main effects of socioeconomic indicators; neither maternal education (*r^2^* = 0.01267, *p* = 0.615) nor income level (*r^2^* = 0.01331, *p* = 0.409) were associated with shifts in the offspring microbial profile. Similarly, neither maternal education nor income were associated with clustering on a PCoA (Fig. S1). Next, the interaction between obesity status with both education (high school graduate or less versus college graduate or more) and income (<$50 k versus ≥$50 k) was examined. An interaction effect between income and obesity status was observed; in the high-income group, a different microbiota community structure was seen in the children of obese versus non-obese mothers (*r^2^* = 0.02547, *p* = 0.041). However, in the lower-income group, no significant effects of maternal obesity on beta diversity were observed (*r^2^* = 0.03798, *p* = 0.139). Also, in dyads from high-income households, the microbiota of children of obese mothers had greater homogeneity among the samples compared to those from non-obese mothers (*F* = 11.942, *p* = 0.003). Furthermore, clustering based on obesity status was observed using a PCoA in the high income group only (Fig. 2A–B).

Similar effects were seen when using education as an indicator of socioeconomic status. Among mothers with a high education, children born to obese mothers had a different community structure than those born to non-obese mothers (*r^2^* = 0.02049, *p* = 0.045) and this was partly explained by significantly greater homogeneity in variance (*F* = 6.215, *p* = 0.02). In contrast, among children born to women with less education, there were no significant differences in beta diversity based on maternal obesity status (*r^2^* = 0.05327, *p* = 0.61). Thus, similar results were observed in relation to income and education as indicators of socioeconomic status. Compared to education level, income was more evenly distributed in the obese and non-obese groups, providing greater statistical power. Thus, all downstream analyses focused on income.

### Maternal obesity and alpha diversity in the child gut microbiome

**Figure 3:**
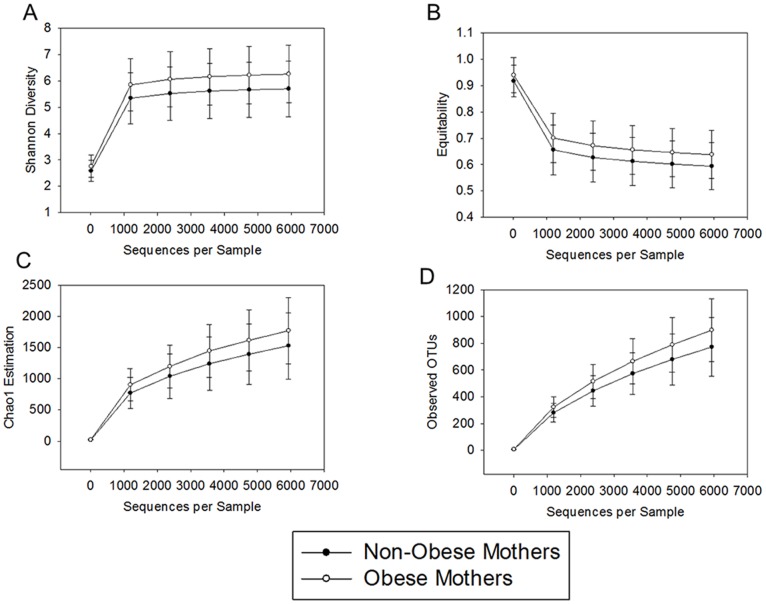
In the overall sample, children born to obese versus non-obese mothers had significantly greater alpha diversity as indicated by A) Shannon Diversity Index (SDI), a measure of overall alpha diversity; B) equitability, a measurement of evenness; C) Chao1, an estimation of richness; and D) the total observed operational taxonomic units (OTUs) (*p*s <.05; Means ±1 SE).

We next examined the relationship between maternal BMI and alpha diversity of the child microbiota. First, we examined the Shannon Diversity Index (SDI), a measure of the overall diversity within a microbial community. Two samples were below the threshold for SDI, resulting in a sample of 75 for these analyses. Results showed that children of obese mothers had a significantly higher SDI than children of non-obese mothers (t(73) = 2.1, *p* = 0.04; Fig. 3A). Greater alpha diversity in children born to obese mothers was associated with greater equitability (t(73) = 1.96, *p* = 0.05; Fig. 3B) and a trend towards greater richness as estimated by Chao1 (t(73) = 1.83, *p* = 0.07; Fig. 3C). Furthermore, children of obese mothers had higher number of unique OTUs as defined by QIIME variable observed_species (t(73) = 2.25, *p* = 0.03; Fig. 3D).

**Figure 4:**
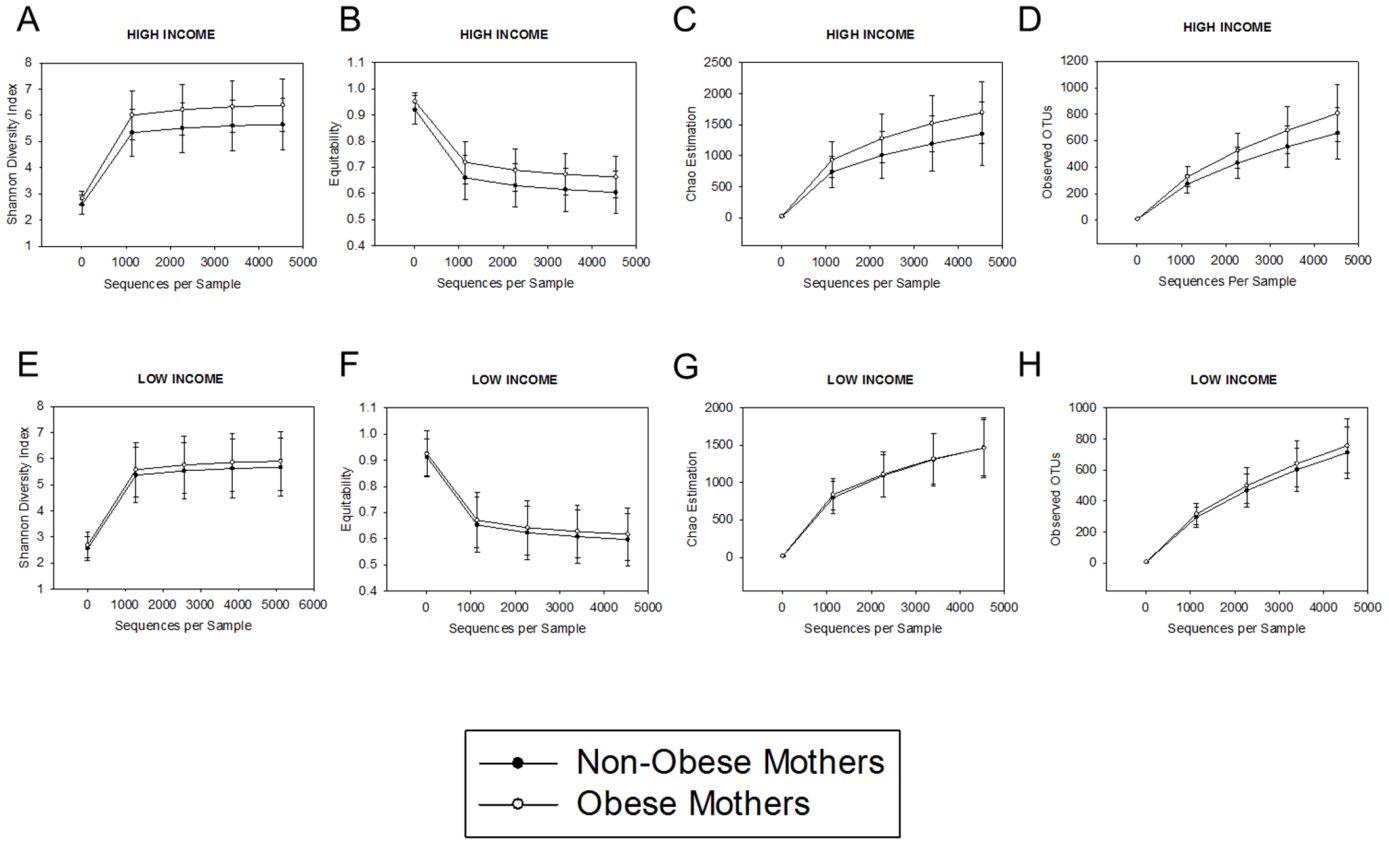
As with measures of beta diversity, differences in alpha diversity in relation to maternal obesity were seen predominately in the higher SES group. In the higher-income group, children born to obese versus non-obese mothers had significantly higher A) Shannon Diversity Index, B) equitability, C) Chao1 estimation, and D) observed operational taxonomic units (OTUs) (*p*s≤0.05). In contrast, in the lower-income group, no significant effects of maternal obesity on alpha diversity indicators were observed (E–H).

Next, we examined interactions between maternal socioeconomic status and obesity on alpha diversity of the child gut microbiome. As with beta diversity, results indicated that effects of maternal obesity on alpha diversity were driven by the high-income group. Specifically, in high income households, SDI (t(73) = 2.30, p = 0.026), Chao1 (t(73) = 2.08, p = 0.043), equitability (t(73) = 2.20, p = 0.033), and observed OTUs (t(73) = 2.30, p = 0.029) were all higher in children of obese versus non-obese mothers. However, among children in lower income households, no differences in alpha diversity were detected in relation to maternal obesity status [SDI (t(73) = 0.537, p = 0.595), Chao1 (t(73) = −0.018, p = 0.992), equitability (t(73) = 0.498, p = 0.619), observed OTUs (t(73) = 0.674, p = 0.515)] (Fig. 4A–H).

Further analyses demonstrated that the SDI was higher in children of obese versus non-obese fathers (t(73) = 1.99, *p* = 0.05) which corresponded to greater equitability (t(73) = 2.10, p = 0.04). However, there were no significant differences in either the Chao1 estimation or OTUs (i.e., observed_species in QIIME) between children born to obese or non-obese fathers (data not shown). When entered into a regression model together, maternal BMI remained a significant predictor of the SDI (β = 0.324, *p = *0.008) while paternal BMI was no longer significantly associated (β = 0.085, *p = *0.48) suggesting that maternal BMI was the critical predictor. In addition, univariate ANOVA demonstrated that the child weight/height ratio showed no association with the toddler SDI (F(2,72) = 0.58, *p* = .565). Moreover, maternal BMI remained a significant predictor after including the child’s WHR in the model (β = 3.178, *p = *0.002), indicating an effect of maternal BMI that was independent of the child’s current body composition.

### Maternal obesity and phylogenetic shifts in child gut microbiome

**Figure 5:**
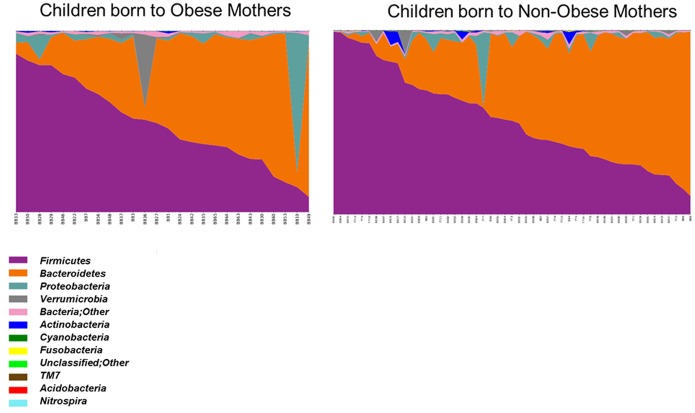
Across individuals, there was considerable variance in the *Firmicutes:Bacteroidetes* ratio, as shown. However, there were no differences between the children born to obese versus non-obese mothers in abundances of the major phyla, *Firmicutes* (*p* = 0.667) and *Bacteroidetes* (*p* = 0.914).

We next examined phylogenetic shifts in the fecal microbiome of the children, to determine if differences in abundances of given genera were evident. An area graph of the phyla present in all subjects indicated that considerable variability existed across children in the abundances of the highly abundant phyla, wherein a wide range of ratios between *Firmicutes:Bacteroidetes* was observed (Fig. 5). Mann-Whitney U-tests revealed no significant differences in the two largest bacterial phyla in the gut, *Firmicutes* (*p* = 0.667) and *Bacteroidetes* (*p* = 0.914) when the relative abundances found in children from obese versus non-obese mothers were compared. When analyses were conducted separately among higher versus lower income groups, no significant effects of maternal obesity on the child gut microbiome at the phyla level were observed that withstood multiple test correction.

Next, genera-level abundances were examined. The Mann-Whitney U test was used due to the skewed distributions of the population abundances. Benjamini-Hochberg tests for multiple comparisons were used, with a q-value set at 0.10. In the overall sample, there were limited significant differences between children born to obese versus non-obese mothers after multiple test correction (Table 3). However, examination of interactions between SES and obesity status revealed multiple associations. Among children of high-income mothers, abundances of the genera *Parabacteroides* (*p* = 0.008, q<0.10), *Eubacterium* (*p* = 0.021, q<0.10), *Blautia* (*p* = 0.025, q<0.10), and *Oscillibacter* (*p* = 0.011, q<0.010), as well as an undefined genus in *Bacteroidales* (*p* = 0.005, q<0.10) differed significantly based on maternal obesity status (Table 4). In contrast, after correction for multiple tests, there were no significant differences between children born to obese versus non-obese mothers in the low-income group (Table 5).

**Table 3:** Top 20 Most Abundant Genera.

	Normal Weight	Obese
*Bacteroides* spp.	36.24±3.76	27.35±4.33
*Lachnospiraceae;* Other	19.31±2.89	13.37±2.44
*Dialister* spp.	5.51±1.67	7.71±2.05
*Faecalibacterium* spp.	5.60±1.46	6.96±2.24*
*Prevotella* spp.	2.85±1.44	9.46±3.94
Unclassified *Clostridiales*	5.22±0.60	4.44±0.73
*Roseburia* spp.	2.97±0.41	4.52±1.21
*Veillonella* spp.	3.31±0.93	2.25±1.62
*Ruminococcaceae*; Other	2.00±0.61	2.42±0.91
*Parabacteroides* spp.	1.78±0.61	2.04±0.57
*Escherichia/Shigella* spp.	1.54±0.79	1.09±0.46
*Alistepes* spp.	1.11±0.34	1.88±0.92
*Ruminococcus* spp.	1.70±0.86	0.61±0.30
Unclassified *Bacteria*	1.09±0.09	1.44±0.17
*Akkermansia* spp.	0.60±0.27	1.88±1.54
*Klebsiella* spp.	0.03±0.02	2.81±2.65
Unclassified *Bacteroidales*	0.67±0.09	0.82±0.11
*Eubacterium* spp.	0.80±0.26	0.34±0.13**
*Oscillibacter* spp.	0.43±0.09	1.00±0.35
*Coprobacillus* spp.	0.28±0.13	1.00±0.81

Data are the mean relative abundance ± standard error.

**p<.05 vs. Non-Obese, passed correction for multiple comparisions.

*p<.05 vs. Non-Obese.

**Table 4:** Top 20 Most Abundant Genera Among High-Income Subjects.

	Normal Weight	Obese
*Bacteroides* spp.	30.65±4.59	31.24±5.86
*Lachnospiraceae;* Other	20.94±3.99	11.13±2.71
*Dialister* spp.	6.85±2.50	5.61±2.66
*Faecalibacterium* spp.	5.83±1.86	5.92±1.64
*Prevotella* spp.	3.06±1.90	11.79±6.71
Unclassified *Clostridiales*	5.85±0.86	3.87±0.66
*Roseburia spp.*	3.68±0.57	6.09±2.12
*Veillonella* spp.	4.55±1.36	3.41±3.00
*Parabacteroides* spp.	1.66±0.91	3.03±0.94**
*Ruminococcaceae* spp.	1.28±0.25	3.10±1.67
*Escherichia/Shigella* spp.	2.24±1.21	0.66±0.34
*Alistepes* spp.	0.93±0.45	2.14±1.52
*Ruminococcus* spp.	1.40±1.02	0.96±0.55
Unclassified *Bacteria*	1.07±0.12	1.36±0.19
*Eubacterium* spp.	0.92±0.38	0.41±0.23**
Unclassified *Bacteroidales*	0.53±0.08	1.01±0.14**
*Akkermansia* spp.	0.70±0.41	0.45±0.26
*Oscillibacte*r spp.	0.28±0.08	1.18±0.60**
*Blautia* spp.	0.53±0.23	0.32±0.25**
Unclassified *Peptostreptococcaceae*	0.53±0.30	0.32±0.22

Data are the mean relative abundance ± standard error.

**p<.05 vs. Non-Obese, passed correction for multiple comparisions.

**Table 5:** Top 20 Most Abundant Genera Among Low-Income Subjects.

	Normal Weight	Obese
*Bacteroides* spp.	46.49±5.97	22.81±6.47*
*Lachnospiraceae;* Other	16.33±3.74	15.99±4.25
*Faecalibacterium* spp.	5.19±2.42	8.19±4.58
*Dialister* spp.	3.05±1.06	10.16±3.14
Unclassified *Clostridiales*	4.06±0.57	5.12±1.39
*Prevotella* spp.	2.46±2.19	6.76±4.79
*Ruminococcaceae;* Other	3.34±1.64	1.64±0.45
*Klebsiella* spp.	0.01±0.00	5.76±5.75
*Roseburia* spp.	1.65±0.35	2.69±0.63
*Akkermansia* spp.	0.42±0.18	3.55±3.33
*Parabacteroides* spp.	1.99±0.49	0.87±0.34
*Alistepes* spp.	1.42±0.52	1.58±1.00
*Ruminococcus* spp.	2.23±1.61	0.20±0.08
Unclassified *Bacteria*	1.13±0.13	1.55±0.30
*Veillonella* spp.	1.03±0.50	0.89±0.41
*Coprobacillus* spp.	0.11±0.05	1.88±1.75
*Escherichia/Shigella* spp.	0.27±0.14	1.60±0.92
Unclassified *Bacteroidales*	0.92±0.19	0.59±0.15
*Oscillibacter* spp.	0.72±0.18	0.78±0.34
*Megasphaera* spp.	1.06±0.85	0.02±0.02

Data are the mean relative abundance ± standard error.

*p<.05 vs. Non-Obese; did not pass correction for multiple comparisons.

### Other behavioral and environmental influences upon the microbiota

In addition to influence by exposure to maternal bacteria, mothers could affect the toddler microbiome via control of the toddler diet, as diet is a primary factor in determining population abundances of the GI microbiota. In chi-square analyses, we found no significant differences in dietary patterns in children of obese versus non-obese women (Table 2). Specifically, children did not differ significantly in duration of breastfeeding, age at which grains/cereals or other foods were introduced, or the frequency of consuming meat or vegetables (*p’s*≥0.15). Children of obese versus non-obese mothers also did not differ in the extent to which they had been exposed to antibiotic medications (during pregnancy, breastfeeding, or directly during childhood) or probiotics in food or supplement form (*p’s*≥ .34).

Because significant results in this study were found predominately in high-income dyads, we further examined potential dietary differences in children born to obese versus non-obese mothers in the high income group. Results also showed no differences in breastfeeding duration, age at which grains/cereals or other foods were introduced, or the frequency of consuming meat or vegetables among children of obese versus non-obese mothers in this group (*p’s*≥0.13).

We also examined the potential role of three key environmental factors that may covary with maternal obesity status and SES: route of delivery (vaginal versus C-section), duration of breastfeeding, and antibiotic exposure in mothers and children. Analyses showed no significant associations between these factors and the community structure of the child gut microbiome (Table S1), and no clustering observed using PCoA (Fig. S2). Also, as described earlier, these exposures did not differ based on maternal obesity status (Table 2). Further analyses among the high-income group also showed that route of delivery, maternal antibiotic use in pregnancy/breastfeeding (combined due to low occurrence), and antibiotic exposure in the child did not differ significantly based on maternal obesity status (*p*s≥ .12).

### Predictive metagenome

The predictive metagenome program, PiCrust, was used to examine if maternal obesity and other factors (duration of breastfeeding, maternal use of antibiotics during breastfeeding or pregnancy, child use of antibiotics, and birth route) were associated with altered functioning of the microbial groups. Abundances of Kyoto Encyclopedia of Genes and Genomes (KEGG) Orthologies, or KOs, were highly similar across children (Fig. S3). Deeper analysis of the KOs revealed that carbohydrate metabolism was significantly lower in children born to obese mothers. However, these differences in KO abundances did not pass correction for multiple tests, due to low effect sizes (Table S2). Likewise, when high and low-income participants were examined separately, maternal obesity was not associated with any significant differences in functional group abundance after multiple test correction (Table S3), nor were differences detected in functional groups based upon breastfeeding duration, antibiotic use by mother or child, and birth route (Tables S4–S6).

## Discussion

Children born to obese mothers are at greater risk for obesity in adulthood compared to children of non-obese mothers, with odds ratios ranging from 1.23 to 6.12 depending on sex and age [3], [37], [38]. Factors including diet and genetics contribute to, but do not fully explain this increased risk [39]. The gut microbiome may play a clinically meaningful role; bacteria that affect metabolic processes are transmitted from the mother to the infant during birth and subsequently through physical contact and, in many cases, breastfeeding [15]–[17]. Obese adults have different microbial community profiles in the gut [9]–[11], and studies show that transplanting microbiota from obese mice into germ-free mice can lead to increased body fat [14], illustrating that altered profiles of microbiota can be both obesogenic and transmittable. However, the extent to which the microbiome may contribute to the intergenerational transmission of obesity in humans is not known.

This study provides novel evidence that maternal obesity is related to measurable differences in the composition of the gut microbiome in offspring, as reflected by measures of both alpha (Shannon Diversity Index, equitability, unique OTUs) and beta diversity (per adonis). Despite the lack of group clustering on a PCoA, differences in beta diversity were explained using permdisp, which indicated increased homogeneity among the microbiomes of the obese-group and increased dispersion among the non-obese group. Our results suggest that the relationship between maternal obesity and the composition of the child gut microbiome remain after accounting for paternal BMI and indicators of child body composition, supporting an exposure rather than purely genetic pathway. This is consistent with epidemiological studies showing that maternal BMI is more strongly associated with obesity in offspring than is paternal BMI [4], [5]. In addition, in metagenome function analyses using PiCRUST, lower abundances of communities related to carbohydrate metabolism were observed in children born to obese versus non-obese mothers, although this result did not remain significant after statistical correction for multiple comparisons.

Importantly, effects of maternal obesity on the composition of the gut microbiome in offspring were stronger and more consistent among those born to mothers of higher socioeconomic status (SES) as defined by income and/or education. Specifically, when higher and lower income groups were examined separately, differences in beta diversity in relation to maternal obesity (per adonis/permdisp and PCoA) were evident only in the higher income group, as were multiple measures of alpha diversity. Less dispersion of profiles among children born to obese compared to non-obese mothers, particularly among those of high SES, indicates that these children are developing microbial profiles typified by greater homogeneity of community structures. Additional studies are needed to determine if similar effects are present in older children, adolescents, and adults.

Also demonstrating effects of socioeconomic status, among the high-income group only, children born to obese versus non-obese mothers had greater abundances of *Parabacteroides* spp., *Oscillibacter* spp., and an unclassified genus of the order *Bacteroidales* as well as lower *Blautia* spp., and *Eubacterium* spp. Of note, differences in *Eubacteriaceae, Oscillibacter* and *Blautia* have been found in prior studies of diet and obesity [40]–[42], but the clinical relevance of these bacterial types in affecting obesity risk is not fully understood. Also, when PiCRUST was used to examine metagemone function based on obesity status in the higher income group only, no significant differences were found.

The mechanisms underlying the interaction between maternal obesity and SES in predicting the composition of the child gut microbiome are not known. Obesity is a health condition with multifactorial origins, both genetic and behavioral (i.e., diet, physical activity). Research on the true interaction between social-environmental and genetic factors (i.e., moderating effects) is sparse. However, among obese adults, the relative contribution of genetic versus behavioral factors may differ in those from higher versus lower socioeconomic backgrounds [43]. Relatedly, the extent to which maternal obesity confers measureable changes to the gut microbiome of offspring may differ based on the etiology of maternal obesity.

Our finding of higher SDI among children of obese versus non-obese mothers contrasts prior research linking obesity with lower alpha diversity [9], [44]. However, previous studies have focused on adults or used mouse models with experimentally-induced obesity. This is one of the first studies to ascertain SDI among toddlers as a function of maternal obesity. Higher SDI in children born to obese mothers may reflect interactions between their unique beta-diversity community profile and age-related effects, possibly down-regulated immune surveillance or reduced GI motility, which could result in greater growth and diversification of microbial groups. Due to the novelty of the study, further investigation is required.

In early life, parents largely control the diet of the child, and tend to offer solid foods that reflect their own adult diets [45]. Diet can substantially affect the composition of the gut microbiome [40], [46], [47]. In our sample, we found no differences in the children from obese and non-obese mothers in terms of breastfeeding behavior, age at which solid foods were introduced, or the current frequency of consumption of meat, vegetables, and cereals/grains regardless of maternal SES. This suggests that diet did not explain the observed differences in the children’s gut microbiome related to maternal obesity and SES. However, this study did not include detailed food diaries that would capture the volume and quality of foods (e.g., high versus low fat meats) consumed. Thus, the possibility remains that differences in feeding behaviors contribute to the observed association with maternal obesity and/or the interaction between maternal obesity and SES.

In addition, other key factors that can affect the gut microbiome including antibiotic exposure, breastfeeding, and route of delivery were examined, but did not account for the observed effects of maternal obesity, or the interaction between maternal obesity and SES. After correction for multiple comparisons, there were not significant differences in individual KOs based upon these factors. Moreover, as described, these factors did not differ significantly based on obesity status, regardless of maternal SES. However, the role of such factors requires further attention.

If continued research supports the notion that obese mothers may pass obesogenic microbiota to their infants, interventions could target manipulation of maternal vaginal and gut microbiome. Prior research has shown that administration of antibiotics during the delivery process reduces vaginal *Lactobacillus* spp. levels in the mother and corresponds to lower levels of lactobacilli in oral samples from newborns [48]. In this case, these effects are potentially detrimental, as early colonization with *Lactobacillus* spp. may have a preventative role in the development of allergic diseases. However, such studies demonstrate that interventions that affect population abundances in the mother can have downstream effects in the neonate’s own microbial structure.

A strength of this study is a focus on children between 18 and 27 months of age. Prior studies have shown that infants of obese mothers have differences in the gut microbiota, specifically the numbers of *Bacteroides* spp. and *Staphylococcus* spp. in the stool [18]. However, the microbiota are characterized by a lack of consistency and high volatility during the first year of life [19]. These profiles generally stabilize and increase in diversity, more closely resembling adult profiles, when the range of dietary exposures for the child expands [20], [49]. Thus, the current data extend prior findings and support the hypothesis that early life exposures may have lasting effects on the gut microbiota. However, considerable variability of the major phyla is still a hallmark of the 18–27 month old child microbiota. In future studies, long-term and longitudinal examination through early childhood and adolescence would be highly valuable in explicating the extent to which observed effects persist and ultimately influence weight.

This study utilized deep pyrosequencing technology which adds upon prior studies by allowing for whole bacterial community profiling of the toddler microbiome. Utilization of this technology allowed increased sensitivity in detecting differences in the gastrointestinal microbiota community structure between children born to obese and non-obese mothers. PiCRUST was used for prediction of metagenome function based upon 16 s rRNA abundances. As reviewed, some effects in relation to maternal obesity were suggested, but these did not remain significant after correction for multiple tests. Unique microbial profiles would be expected to result in differences in microbiome function. True metagenomic shotgun sequencing will likely provide greater power to examine effects of factors such as maternal obesity on the function of the microbiota in children.

In this study, parental BMI as well as children’s body composition indicators (height and weight percentile) were collected via maternal report rather than direct measurement. Current maternal BMI was not the focus because 1) maternal BMI may have changed considerably since the target pregnancy (e.g., due to weight retention after the target pregnancy or subsequent weight gain) and 2) women were of childbearing age, thus a meaningful proportion were pregnant with another child at the time of data collection. Prior studies suggest that among women of reproductive age, BMI classified by self-reported height and weight is generally accurate, resulting in correct categorization of 84%–87% and an underestimate in BMI of 0.8 kg/m^2^ [50], [51]. Because BMI by self-report tends to be slightly lower than true BMI, effects of maternal obesity on outcomes of interest may be underestimated in the current study. In addition, this study did not include collection of maternal specimens, such as vaginal or fecal samples, which would permit profiling of maternal microbial communities. This is clearly a critical next step in establishing a direct link from maternal to child microbial profiles.

In conclusion, obesity is a worldwide public health issue. Identification of modifiable early life antecedents is key to addressing this disease process. A rapidly growing body of literature indicates that the gut microbiome plays a critical role in the development of obesity. Adding to this literature, the current study provides novel evidence that maternal obesity is associated with different microbial profiles in offspring 18–27 months of age. The potential role of the gut microbiome in this intergenerational transmission of obesity risk warrants further attention. In particular, the stability of such effects into later childhood and adolescence, the clinical relevance of abundances of specific bacteria in conferring risk for obesity, and the ultimate impact of early life microbial profiles on long-term weight trajectory remains to be explicated.

## Supporting Information

Figure S1 **Indicators of socioeconomic status (SES), maternal education (A) and income (B) did not predict differences in the offspring microbiota community structure.**
(TIF)

Figure S2 **Other key factors which may impact the gut microbiome were not associated with differences in community structure, including (A) birth route (B) antibiotic use by the mother while breastfeeding (C) antibiotic use during pregnancy (D), child antibiotic use or (E) duration of breastfeeding.**
(TIF)

Figure S3 **KEGG Orthologues (KOs) were highly similar across individuals.** PiCRUST was used to predict metagenomic function of the child microbiome. An area graph produced by QIIME indicated that overall abundances of KOs were similar across samples.
(TIF)

Table S1 **Potential Impacts Upon the Offspring Microbiota.**
(DOC)

Table S2 **KEGG Orthologues.**
(DOCX)

Table S3 **KEGG Orthologues among High-Income Subjects.**
(DOCX)

Table S4 **KEGG Orthologues.**
(DOCX)

Table S5 **KEGG Orthologues.**
(DOCX)

Table S6 **KEGG Orthologues.**
(DOCX)
