# Supplementary figures and images for: Maternal Obesity Is Associated with Alterations in the Gut Microbiome in Toddlers
Source: PLoS One. 2014 Nov 19;9(11):e113026. doi: 10.1371/journal.pone.0113026 (PMC4237395; doi:10.1371/journal.pone.0113026)

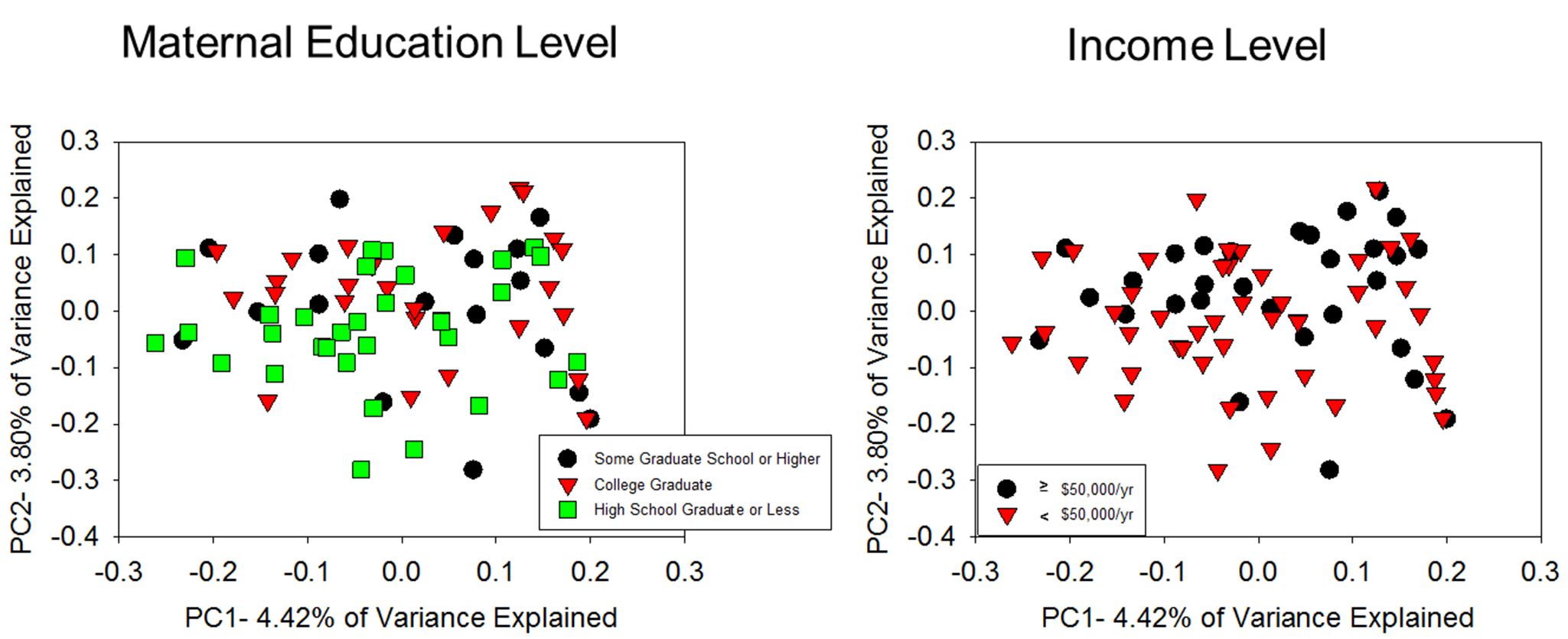

Supplement: Figure S1 — Indicators of socioeconomic status (SES), maternal education (A) and income (B) did not predict differences in the offspring microbiota community structure. (TIF) [file pone.0113026.s001.tif]

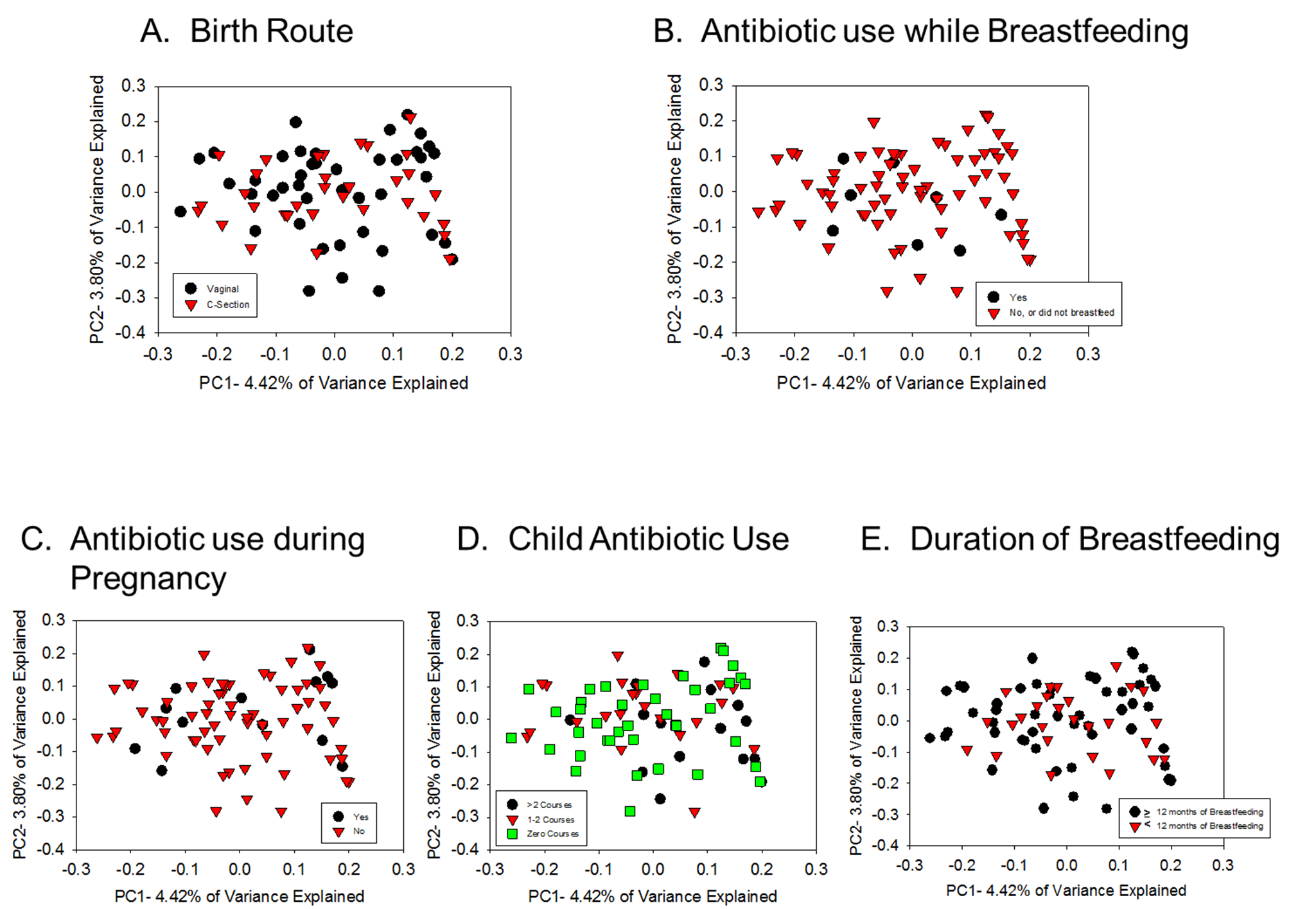

Supplement: Figure S2 — Other key factors which may impact the gut microbiome were not associated with differences in community structure, including (A) birth route (B) antibiotic use by the mother while breastfeeding (C) antibiotic use during pregnancy (D), child antibiotic use or (E) duration of breastfeeding. (TIF) [file pone.0113026.s002.tif]

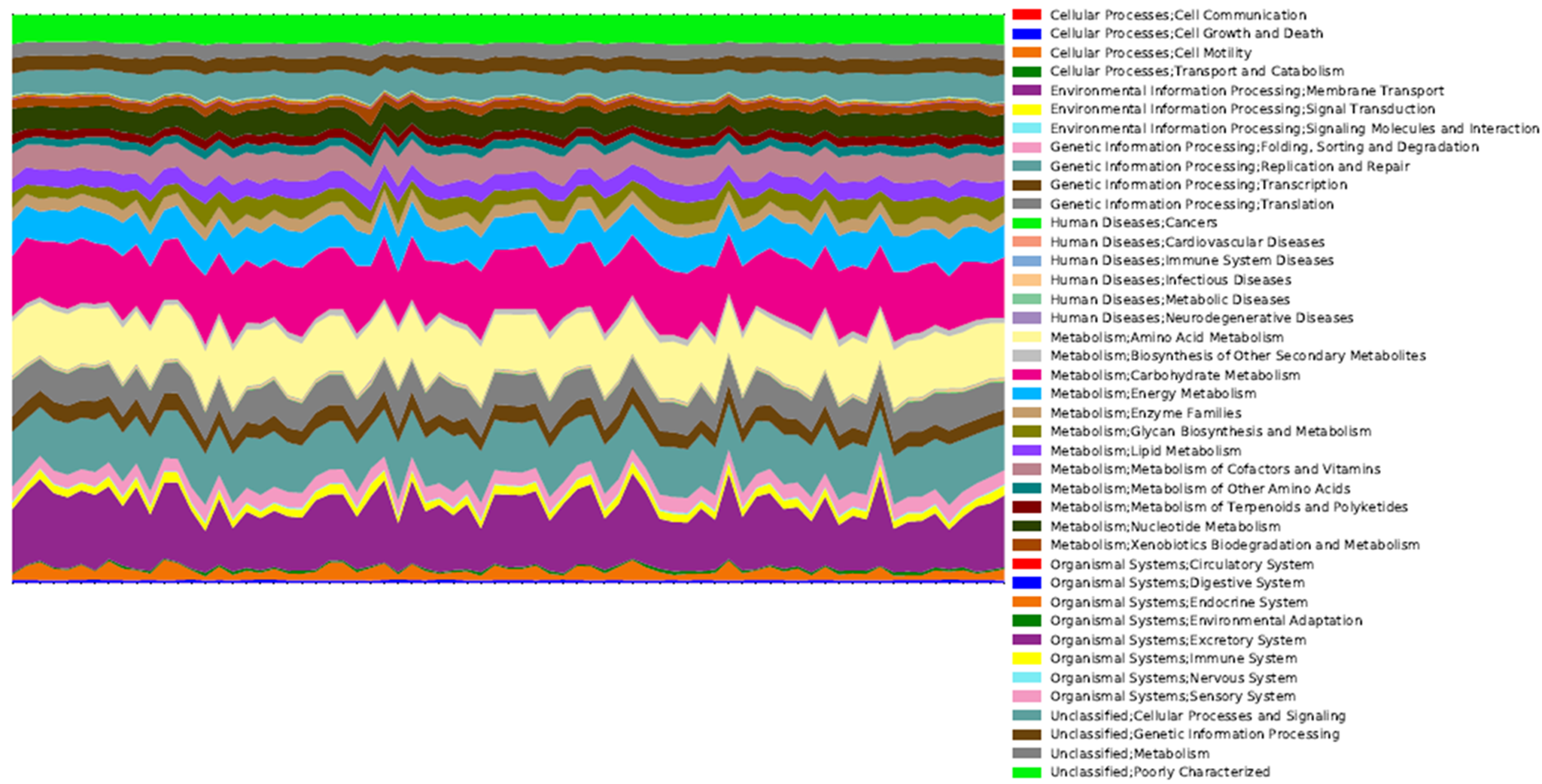

Supplement: Figure S3 — KEGG Orthologues (KOs) were highly similar across individuals. PiCRUST was used to predict metagenomic function of the child microbiome. An area graph produced by QIIME indicated that overall abundances of KOs were similar across samples. (TIF) [file pone.0113026.s003.tif]
