# Supplementary material for: Maternal Obesity Is Associated with Alterations in the Gut Microbiome in Toddlers
Source: PLoS One. 2014 Nov 19;9(11):e113026. doi: 10.1371/journal.pone.0113026 (PMC4237395; doi:10.1371/journal.pone.0113026)
Supplement: Table S1 — Potential Impacts Upon the Offspring Microbiota. (DOC) [file pone.0113026.s004.doc]

Table S1: Potential Impacts Upon the Offspring Microbiota

|  | P-value | R^2^ |
| --- | --- | --- |
| Birth route (vaginal vs. caesarean) | 0.661 | 0.0126 |
| Breastfeeding duration (>12 months vs. <12 months) | 0.714 | 0.0125 |
| Mother took antibiotics during pregnancy (yes vs. no) | 0.994 | 0.0109 |
| Mother took antibiotics while breastfeeding (yes vs. no) | 0.363 | 0.0134 |
| Child has taken antibiotics (0 courses vs. 1-2 courses vs. >2 courses) | 0.926 | 0.0116 |

Data are the calculated p-value and R2 (effect size) as measured with adonis.
