# Supplementary material for: Maternal Obesity Is Associated with Alterations in the Gut Microbiome in Toddlers
Source: PLoS One. 2014 Nov 19;9(11):e113026. doi: 10.1371/journal.pone.0113026 (PMC4237395; doi:10.1371/journal.pone.0113026)
Supplement: Table S2 — KEGG Orthologues. (DOCX) [file pone.0113026.s005.docx]

Table S2. KEGG Orthologues

|  | Non-Obese | Obese |
| --- | --- | --- |
| Membrane Transport | 11.33 ± 2.23 | 11.16 ± 2.23 |
| Carbohydrate Metabolism | 11.13 ± 0.78 | 10.70 ± 0.83 |
| Amino Acid Metabolism | 9.73 ± 0.30 | 9.71 ± 0.29 |
| Replication and Repair | 8.71 ± 0.52 | 8.84 ± 0.67 |
| Energy Metabolism | 5.91 ± 0.32 | 5.88 ± 0.35 |
| Translation | 5.42 ± 0.47 | 5.57 ± 0.51 |
| Metabolism of Cofactors and Vitamins | 4.51 ± 0.32 | 4.57 ± 0.33 |
| Cellular Processes and Signaling | 4.42 ± 0.30 | 4.43 ± 0.24 |
| Nucleotide Metabolism | 4.02 ± 0.26 | 4.11 ± 0.35 |
| Lipid Metabolism | 2.93 ± 0.18 | 2.93 ± 0.21 |
| Glycan Biosynthesis and Metabolism | 2.89 ± 0.82 | 2.93 ± 0.71 |
| Transcription | 2.79 ± 0.27 | 2.74 ± 0.23 |
| Genetic Information Processing | 2.55 ± 0.19 | 2.62 ± 0.16 |
| Folding, Sorting and Degradation | 2.48 ± 0.18 | 2.52 ± 0.20 |
| Metabolism | 2.53 ± 0.17 | 2.47 ± 0.15 |
| Enzyme Families | 2.18 ± 0.08 | 2.21 ± 0.12 |
| Cell Motility | 1.77 ± 0.79 | 1.81 ± 0.75 |
| Metaoblism of Terpenoids and Polyketides | 1.63 ± 0.11 | 1.66 ± 0.15 |
| Metabolism of Other Amino Acids | 1.53 ± 0.14 | 1.56 ± 0.12 |
| Xenobiotics Biodegradation and Metabolism | 1.50 ± 0.15 | 1.54 ± 0.31 |
| Signal Transduction | 1.49 ± 0.21 | 1.50 ± 0.26 |
| Biosynthesis of Other Secondary Metabolites | 1.01 ± 0.16 | 1.00 ± 0.13 |
| Cell Growth and Death | 0.50 ± 0.04 | 0.51 ± 0.05 |
| Transport and Catabolism | 0.40 ± 0.16 | 0.39 ± 0.12 |
| Signaling Molecuels and Interaction | 0.20 ± 0.05 | 0.19 ± 0.04 |
| Environmental Adaptation | 0.16 ± 0.03 | 0.16 ± 0.02 |

Data are KEGG Orthologue mean relative frequency (in %) ± standard deviation
