# Supplementary material for: Maternal Obesity Is Associated with Alterations in the Gut Microbiome in Toddlers
Source: PLoS One. 2014 Nov 19;9(11):e113026. doi: 10.1371/journal.pone.0113026 (PMC4237395; doi:10.1371/journal.pone.0113026)
Supplement: Table S3 — KEGG Orthologues among High-Income Subjects. (DOCX) [file pone.0113026.s006.docx]

.

Table S3. KEGG Orthologues among High-Income Subjects

|  | Non-Obese | Obese |
| --- | --- | --- |
| Membrane Transport | 11.22 ± 2.25 | 11.25 ± 2.30 |
| Carbohydrate Metabolism | 11.26 ± 0.66 | 11.03 ± 0.69 |
| Amino Acid Metabolism | 9.73 ± 0.34 | 9.74 ± 0.29 |
| Replication and Repair | 8.68 ± 0.52 | 8.80 ± 0.52 |
| Energy Metabolism | 5.97 ± 0.27 | 5.96 ± 0.25 |
| Translation | 5.40 ± 0.43 | 5.50 ± 0.44 |
| Metabolism of Cofactors and Vitamins | 4.48 ± 0.27 | 4.52 ± 0.33 |
| Cellular Processes and Signaling | 4.44 ± 0.28 | 4.42 ± 0.22 |
| Nucleotide Metabolism | 4.01 ± 0.23 | 4.06 ± 0.26 |
| Lipid Metabolism | 2.94 ± 0.18 | 2.91 ± 0.15 |
| Glycan Biosynthesis and Metabolism | 2.87 ± 0.81 | 2.83 ± 0.73 |
| Transcription | 2.83 ± 0.25 | 2.80 ± 0.24 |
| Genetic Information Processing | 2.52 ± 0.13 | 2.57 ± 0.12 |
| Metabolism | 2.54 ± 0.18 | 2.49 ± 0.15 |
| Folding, Sorting, and Degradation | 2.51 ± 0.19 | 2.40 ± 0.19 |
| Enzyme Families | 2.20 ± 0.08 | 2.21 ± 0.06 |
| Cell Motility | 1.70 ± 0.74 | 1.74 ± 0.70 |
| Metabolism of Terpenoids and Polyketides | 1.64 ± 0.12 | 1.63 ± 0.12 |
| Metabolism of Other Amino Acids | 1.54 ± 0.14 | 1.54 ± 0.11 |
| Xenobiotics Biodegradation and Metabolism | 1.50 ± 0.15 | 1.49 ± 0.11 |
| Signal Transduction | 1.49 ± 0.20 | 1.48 ± 0.19 |
| Biosynthesis of Other Secondary Metabolites | 1.03 ± 0.16 | 1.03 ± 0.12 |
| Cell Growth and Death | 0.50 ± 0.04 | 0.51 ± 0.04 |
| Transport and Catabolism | 0.40 ± 0.16 | 0.39 ± 0.14 |
| Signaling Molecules and Interaction | 0.20 ± 0.05 | 0.19 ± 0.05 |
| Environmental Adaptation | 0.16 ± 0.02 | 0.16 ± 0.02 |

Data are from KEGG Orthologues mean relative frequency (in %) ± standard deviation
