# Supplementary material for: Maternal Obesity Is Associated with Alterations in the Gut Microbiome in Toddlers
Source: PLoS One. 2014 Nov 19;9(11):e113026. doi: 10.1371/journal.pone.0113026 (PMC4237395; doi:10.1371/journal.pone.0113026)
Supplement: Table S4 — KEGG Orthologues. (DOCX) [file pone.0113026.s007.docx]

Table S4. KEGG Orthologues

|  | Less than 12 months Breastfeeding | 12 Months or Greater Breastfeeding |
| --- | --- | --- |
| Carbohydrate Metabolism | 10.96 ± 0.86 | 11.22 ± 0.71 |
| Membrane Transport | 11.20 ± 2.15 | 10.72 ± 2.25 |
| Amino Acid Metabolism | 9.72 ± 0.30 | 9.79 ± 0.25 |
| Replication and Repair | 8.76 ± 0.60 | 8.83 ± 0.49 |
| Energy Metabolism | 5.87 ± 0.33 | 6.08 ± 0.26 |
| Translation | 5.47 ± 0.50 | 5.43 ± 0.46 |
| Metabolism of Cofactors and Vitamins | 4.53 ± 0.34 | 4.58 ± 0.29 |
| Cellular Processes and Signaling | 4.45 ± 0.28 | 4.42 ± 0.28 |
| Nucleotide Metabolism | 4.05 ± 0.30 | 4.09 ± 0.27 |
| Glycan Biosynthesis and Metabolism | 2.97 ± 0.75 | 2.90 ± 0.86 |
| Lipid Metabolism | 2.93 ± 0.20 | 2.92 ± 0.15 |
| Transcription | 2.76 ± 0.25 | 2.75 ± 0.29 |
| Genetic Information Processing | 2.58 ± 0.19 | 2.50 ± 0.09 |
| Folding, Sorting, and Degradation | 2.50 ± 0.18 | 2.57 ± 0.20 |
| Metabolism | 2.52 ± 0.16 | 2.49 ± 0.18 |
| Enzyme Families | 2.18 ± 0.10 | 2.23 ± 0.07 |
| Cell Motility | 1.77 ± 0.78 | 1.79 ± 0.80 |
| Metabolism of Terpenoids and Polyketides | 1.64 ± 0.12 | 1.67 ± 0.12 |
| Metabolism of Other Amino Acids | 1.55 ± 0.14 | 1.54 ± 0.12 |
| Xenobiotics Biodegradation and Metabolism | 1.52 ± 0.23 | 1.47 ± 0.10 |
| Signal Transduction | 1.50 ± 0.23 | 1.46 ± 0.18 |
| Biosynthesis of Other Secondary Metabolites | 1.01 ± 0.16 | 1.01 ± 0.10 |
| Cell Growth and Death | 0.51 ± 0.05 | 0.50 ± 0.04 |
| Transport and Catabolism | 0.40 ± 0.15 | 0.40 ± 0.16 |
| Signaling Molecules and Interaction | 0.20 ± 0.05 | 0.20 ± 0.06 |
| Environmental Adaptation | 0.16 ± 0.02 | 0.16 ± 0.03 |

Data are from KEGG Orthologue mean relative frequency (in %) ± standard deviation
