# Supplementary material for: Maternal Obesity Is Associated with Alterations in the Gut Microbiome in Toddlers
Source: PLoS One. 2014 Nov 19;9(11):e113026. doi: 10.1371/journal.pone.0113026 (PMC4237395; doi:10.1371/journal.pone.0113026)
Supplement: Table S5 — KEGG Orthologues. (DOCX) [file pone.0113026.s008.docx]

Table S5. KEGG Orthologues

|  | Vaginal | Caesarean |
| --- | --- | --- |
| Membrane Transport | 11.32 ± 2.26 | 11.21 ± 2.20 |
| Carbohydrate Metabolism | 10.92 ± 0.88 | 11.08 ± 0.73 |
| Amino Acid Metabolism | 9.75 ± 0.26 | 9.69 ± 0.33 |
| Replication and Repair | 8.75 ± 0.57 | 8.76 ± 0.59 |
| Energy Metabolism | 5.89 ± 0.35 | 5.92 ± 0.30 |
| Translation | 5.46 ± 0.49 | 5.48 ± 0.49 |
| Metabolism of Cofactors and Vitamins | 4.56 ± 0.33 | 4.49 ± 0.32 |
| Cellular Processes and Signaling | 4.42 ± 0.29 | 4.43 ± 0.27 |
| Nucleotide Metabolism | 4.04 ± 0.28 | 4.06 ± 0.31 |
| Lipid Metabolism | 2.92 ± 0.20 | 2.94 ± 0.18 |
| Glycan Biosynthesis and Metabolism | 2.91 ± 0.77 | 2.91 ± 0.80 |
| Transcription | 2.76 ± 0.26 | 2.79 ± 0.24 |
| Genetic Information Processing | 2.57 ± 0.19 | 2.57 ± 0.17 |
| Folding, Sorting, and Degradation | 2.50 ± 0.17 | 2.52 ± 0.15 |
| Metabolism | 2.50 ± 0.20 | 2.49 ± 0.17 |
| Enzyme Families | 2.18 ± 0.09 | 2.19 ± 0.11 |
| Cell Motility | 1.81 ± 0.79 | 1.75 ± 0.76 |
| Metabolism of Terpenoids and Polyketides | 1.63 ± 0.13 | 1.64 ± 0.11 |
| Metabolism of Other Amino Acids | 1.54 ± 0.13 | 1.54 ± 0.15 |
| Xenobiotics Biodegradation and Metabolism | 1.52 ± 0.25 | 1.50 ± 0.17 |
| Signal Transduction | 1.51 ± 0.23 | 1.48 ± 0.21 |
| Biosynthesis of Other Secondary Metabolites | 1.00 ± 0.15 | 1.02 ± 0.15 |
| Cell Growth and Death | 0.51 ± 0.05 | 0.50 ± 0.04 |
| Transport and Catabolism | 0.39 ± 0.15 | 0.39 ± 0.14 |
| Signaling Molecules and Interaction | 0.19 ± 0.05 | 0.20 ± 0.04 |
| Environmental Adaptation | 0.16 ± 0.02 | 0.16 ± 0.02 |

Data are from KEGG Orthologue mean relative frequency (in %) ± standard deviation
