# Supplementary material for: Maternal Obesity Is Associated with Alterations in the Gut Microbiome in Toddlers
Source: PLoS One. 2014 Nov 19;9(11):e113026. doi: 10.1371/journal.pone.0113026 (PMC4237395; doi:10.1371/journal.pone.0113026)
Supplement: Table S6 — KEGG Orthologues. (DOCX) [file pone.0113026.s009.docx]

Table S6. KEGG Orthologues

|  | Zero Courses | 1-2 Courses | Over 2 Courses |
| --- | --- | --- | --- |
| Membrane Transport | 11.73 ± 2.32 | 10.30 ± 1.94 | 11.37 ± 2.17 |
| Carbohydrate Metabolism | 10.93 ± 0.83 | 11.40 ± 0.67 | 10.88 ± 0.82 |
| Amino Acid Metabolism | 9.73 ± 0.27 | 9.78 ± 0.23 | 9.70 ± 0.33 |
| Replication and Repair | 8.70 ± 0.58 | 8.81 ± 0.58 | 8.76 ± 0.58 |
| Energy Metabolism | 5.89 ± 0.32 | 5.99 ± 0.28 | 5.87 ± 0.35 |
| Translation | 5.48 ± 0.47 | 5.38 ± 0.44 | 5.50 ± 0.51 |
| Metabolism of Cofactors and Vitamins | 4.49 ± 0.33 | 4.51 ± 0.28 | 4.56 ± 0.33 |
| Cellular Processes and Signaling | 4.37 ± 0.30 | 4.52 ± 0.27 | 4.42 ± 0.26 |
| Nucleotide Metabolism | 3.99 ± 0.29 | 4.67 ± 0.28 | 4.07 ± 0.29 |
| Lipid Metabolism | 2.95 ± 0.19 | 2.98 ± 0.14 | 2.90 ± 0.20 |
| Glycan Biosynthesis and Metabolism | 2.68 ± 0.87 | 3.11 ± 0.74 | 2.96 ± 0.71 |
| Transcription | 2.82 ± 0.28 | 2.75 ± 0.21 | 2.75 ± 0.25 |
| Genetic Information Processing | 2.56 ± 0.18 | 2.53 ± 0.11 | 2.59 ± 0.20 |
| Metabolism | 2.47 ± 0.16 | 2.55 ± 0.14 | 2.52 ± 0.17 |
| Folding, Sorting, and Degradation | 2.47 ± 0.20 | 2.56 ± 0.17 | 2.49 ± 0.18 |
| Enzyme Families | 2.17 ± 0.09 | 2.23 ± 0.11 | 2.18 ± 0.09 |
| Cell Motility | 1.93 ± 0.84 | 1.59 ± 0.58 | 1.77 ± 0.79 |
| Metabolism of Terpenoids and Polyketides | 1.62 ± 0.15 | 1.69 ± 0.11 | 1.63 ± 0.12 |
| Metabolism of Other Amino Acids | 1.52 ± 0.16 | 1.56 ± 0.14 | 1.54 ± 0.12 |
| Xenobiotics Biodegradation and Metabolism | 1.55 ± 0.34 | 1.51 ± 0.14 | 1.50 ± 0.14 |
| Signal Transduction | 1.54 ± 0.25 | 1.44 ± 0.15 | 1.49 ± 0.23 |
| Biosynthesis of Other Secondary Metabolites | 0.98 ± 0.15 | 1.07 ± 0.13 | 1.00 ± 0.15 |
| Cell Growth and Death | 0.51 ± 0.05 | 0.49 ± 0.04 | 0.51 ± 0.05 |
| Transport and Catabolism | 0.36 ± 0.15 | 0.45 ± 0.14 | 0.39 ± 0.14 |
| Signaling Molecules and Interaction | 0.18 ± 0.06 | 0.21 ± 0.04 | 0.20 ± 0.04 |
| Environmental Adaptation | 0.17 ± 0.03 | 0.15 ± 0.02 | 0.16 ± 0.02 |

Data are from KEGG Orthologue mean relative frequency (in %) ± standard deviation
